# Management of Oral Mucositis in Children With Malignant Solid Tumors

**DOI:** 10.3389/fonc.2021.599243

**Published:** 2021-03-30

**Authors:** Giorgio Attinà, Alberto Romano, Palma Maurizi, Sara D’Amuri, Stefano Mastrangelo, Michele Antonio Capozza, Silvia Triarico, Antonio Ruggiero

**Affiliations:** ^1^ Unità di Oncologia Pediatrica, Fondazione Policlinico Universitario Gemelli IRCCS, Università Cattolica del Sacro Cuore, Rome, Italy; ^2^ Unità di Oncologia, Azienda Ospedaliera Sant’Andrea, Rome, Italy

**Keywords:** children, cancer, mucositis, chemotherapy, pain

## Abstract

**Introduction:**

In recent years, the use of intensive regimens for the treatment of pediatric cancer has led to a marked improvement in patient survival. However, these treatments are associated with an increase in toxic effects. Among these side effects, mucositis (inflammation of the oral cavity) significantly affect the success of treatment. The aim of this study was to assess the prevalence of mucositis in a pediatric population with solid tumor and undergoing chemotherapy, identify the risk factors that influence its occurrence, and verify the usefulness of pain rating scales.

**Methods:**

We registered episodes of mucositis which occurred in a sample of 84 consecutive children with solid tumors between 1 January, 2012 and 30 April, 2018. The World Health Organization (WHO) oral mucositis grading scale and the modified Wong–Baker FACES Pain Rating Scale (WBS) were used to assess the severity of each episode. Moreover, data on the treatments used and blood count results were collected.

**Results:**

The prevalence of mucositis in our population was 50%, without statistically significant difference according to sex and a higher prevalence observed in patients aged >10 years. The presence of neutropenia, higher number of cycles of chemotherapy, and co-existence of lymphomas and sarcomas were identified as factors favoring the occurrence of mucositis. The WBS showed results superimposed on the WHO oral mucositis grading scale in choosing the intensity and duration of mucositis treatment.

**Conclusion:**

Oral mucositis is a common complication of chemotherapy against childhood malignancies. The WHO oral mucositis scale is a valuable tool for assessing its severity in pediatric patients. Furthermore, WBS can be used as an assessment tool to establish the therapy to be adopted for patients in whom direct evaluation of the oral cavity is not possible.

## Introduction

Pediatric cancers are rare diseases, although their incidence has increased in recent years. However, the cumulative overall survival is increased due to the adoption of international cooperative treatment regimens, which permit the combination of surgery, radiotherapy and chemotherapy. In parallel with the increase in survival, increased toxicity has been observed both at the hematological and extra-hematological levels (1–5).

Along with nausea and vomiting (6), mucositis is one of the most frequent side effects associated with the treatment of pediatric cancers; it is an inflammation of the mucosa of the oral cavity with multiple etiologies causing pain and inability to eat. Lack of control and inadequate prevention of mucositis can lead to a significant decline in patient quality of life (i.e., pain, difficulty feeding, and malnutrition) (7–9).

The incidence rate of oral mucositis ranges from 52% to 100% of patients receiving high-dose chemotherapy. If not managed with adequate measures, mucositis represents an important limiting factor of chemotherapy and may worsen patient prognosis and compliance (10–12).

Appropriate oral hygiene represents the main intervention in children receiving chemotherapy (13, 14). In addition, in the presence of mucositis, a systematic pain assessment allows the selection of appropriate treatment and reduces the rate of treatment-related side effects. Although there are numerous publications on the prevention and treatment of oral mucositis, a specific standard treatment protocol for children with cancer is currently unavailable (15).

The main objective of this study was to evaluate the prevalence of mucositis in a pediatric population, identify the risk factors that influence its occurrence, and verify the usefulness of pain rating scales. The secondary objective of the study was to verify the effectiveness of the pain treatment routinely adopted at our center.

## Methods

Our study assessed 84 consecutive children with solid tumors treated in the Paediatric Oncology Unit of Agostino Gemelli Hospital (Rome, Italy) between 1 January 2012 and 30 April 2018. Inclusion criteria were: (a) age 4–18 years; (b) diagnosis of solid tumor; (c) administration of at least one course of chemotherapy during the study period; (d) no concomitant radiation therapy; and (e) no surgery on the head region, face, and digestive system.

Of 84 patients examined, 33 were affected by central nervous system tumors, 23 patients by sarcomas, 11 patients by lymphomas and 17 patients by other solid tumors (mainly neuroblastomas, nephroblastomas, and retinoblastomas).

Patients with leukemia were not included in the study due to the different type of treatment they undergo. Patients with solid tumors receive chemotherapy courses every three to four weeks while patients with leukemia receive chemotherapy treatments with shorter timescales. For this reason, in our study we did not consider these two categories of patients to be comparable.

All patients were given an oral hygiene protocol based on the use of a soft toothbrush for patients with a platelet count <50,000 cells/ml, or a sponge brush or a wet gauze for patients with a platelet count <20,000 cells/ml, and prophylactic oral rinsing/swabbing with alcohol-free 0.2% chlorhexidine oral rinse for four times a day (16).

If mucositis was diagnosed, the World Health Organization (WHO) oral mucositis grading scale was adopted to evaluate its severity (17) (**Table 1**).

**Table 1:** The WHO oral mucositis grading scale (17).

Grade	Description
0 (none)	None
I (mild)	Oral sorenes,erythema
II (moderate)	Oral erythema, ulcers, solid diet tolerated
III (severe)	Oral ulcers, liquid diet only
IV (life-threatening)	Oral alimentation impossible

In addition, all patients underwent evaluation using a Pain Rating Scale. We adopted the modified Wong–Baker FACES Pain Rating Scale (WBS) with a score between 0 and 5 (18). This evaluation was performed thrice daily.

In order to be able to compare the two scales, we excluded patients under the age of 4 due to the possible difficulty in carrying out a complete evaluation of the oral cavity as required by the WHO oral mucositis grading scale.

All children who were admitted and had received chemotherapy within the past 14 days were considered neutropenic when the neutrophil count was <500 cells/ml.

Based on the intensity of pain and duration of symptoms, pain relief treatment was initiated with pethidine through continuous intravenous infusion at a dosage of 2 mg/kg/day. Clonazepam was orally administered in combination (1 drop/10 kg/day) to prevent the risk of seizures associated with pethidine. If treatment with pethidine was ineffective against pain, fentanyl was administered intravenously through continuous infusion at the initial dosage of 0.5 µg/kg/h. All patients underwent multiparametric monitoring during treatment. Finally, all patients with evident inability to eat underwent intravenous parenteral nutrition.

Data are presented as the mean and standard deviation (SD). The Kruskal–Wallis test was used for comparison of values between groups. The chi-squared test was used for comparison of proportions. The Spearmen’s linear correlation coefficient (r) was used to study the relationship between continuous variables. A p-value <0.05 denoted statistically significant differences.

The patients evaluated were not subjected to treatments or procedures other than those required by the mucositis management protocols approved by our facility. Prior to the data analysis, the caregivers provided signed consent forms after being informed about the aim of the project as foreseen by the Italian Law on Privacy and Safeguarding of Sensitive Data (D. Lgs n196,2003). In the text there are no data that can be traced back specifically to any patient.

## Results

84 patients were selected (mean age: 13.1 years [SD: 5.6]; 43 males [51%]; 41 females [49%]). Of those, 42 patients had at least one episode of mucositis, that is 50% of the population, {mean age: 13.6 years [SD: 5.2]; 19 females (45.2%); 23 males (54.8%); 33 (79%) and nine (21%) patients were aged >10 and <10 years, respectively}. There was no difference in the incidence of mucositis in relation to sex (p = 0.9), while the incidence was higher in patients aged >10 years (p < 0.001). However, a relationship was not identified between the number of episodes and age of patients (r = 0.15; p = 0.18).

**Figure 1:**
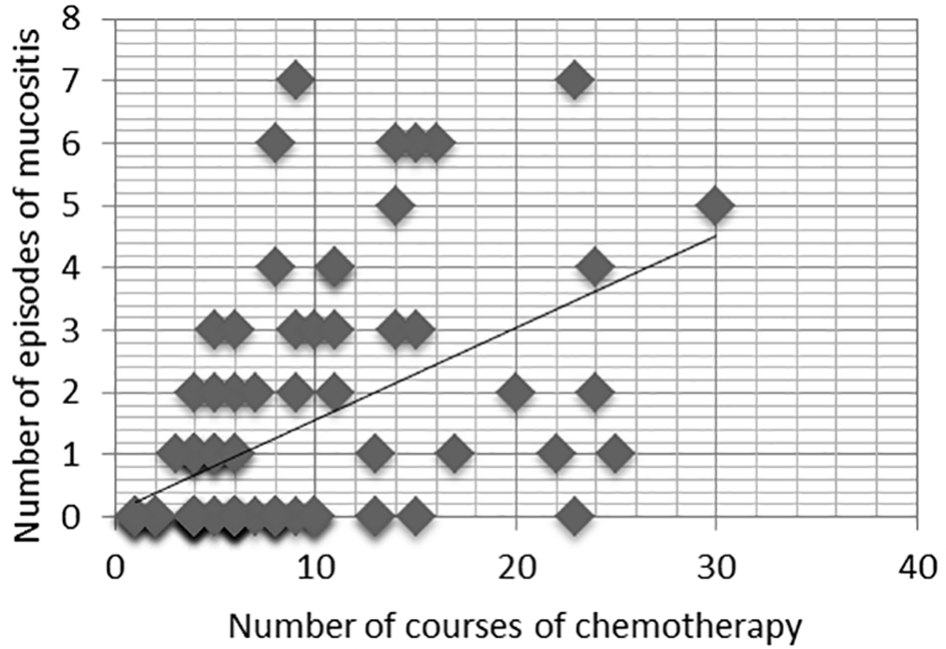
Relationship between the number of courses of chemotherapy and the number of episodes of mucositis, r=0.52, p< 0.01.

The number of episodes of mucositis caused by chemotherapy varied in the study population. Of the 42 patients, 16 had one episode (38.1%), seven patients had two episodes (16.7%), seven patients had three episodes (16.7%), four patients had four episodes (9.5%), two patients had five episodes (4.8%), four patients had six episodes (9.5%), and only two patients had seven total events (4.8%) throughout the duration of the drug treatment. We identified an increasing linear relationship between the number of episodes of mucositis and the number of cycles of chemotherapy administered (r = 0.52; p < 0.01) (**Figure 1**).

The time of onset of mucositis was measured in days, starting on day 0 as the first day of chemotherapy. The occurrence of mucositis has been observed from a minimum of 5 days to a maximum of 17 days (mean: 10.4 days [SD: 3.6]). The duration of mucositis ranged 2–20 days (mean: 7.3 days [SD: 4.89]).

Patients were stratified according to the diagnosis into four groups: (1) patients with central nervous system tumors; (2) patients with sarcomas; (3) patients with lymphomas; and (4) patients with solid tumors, mainly neuroblastomas, nephroblastomas, and retinoblastomas. Mucositis events were also analyzed by following their distribution among the same subgroups used for the classification of patients. **Table 2** summarizes the characteristics of patients and episodes of mucositis stratified by the types of tumors.

**Table 2:** Characteristics of the children stratified by tumor’s type.

	Number of patients	Average age (years)	Number of CT cycles	Average number of CT cycles/patients
**CNS Tumor**	33	11,8	169	5
**Sarcomas**	23	15,1	334	13
**Lymphomas**	11	18	110	6
**Other**	17	7,7	162	6
**p**		**0,01**		**0,01**
	**Number of patients with mucositis**	**Total number of episode of mucositis observed**	**Average number of CT cycles with mucositis** **/patients**	**Percentage of cycles of CT with mucositis (%)**
**CNS Tumor**	6	10	1,7	6
**Sarcomas**	18	56	3	17
**Lymphomas**	6	21	3,5	19
**Other**	12	28	2,3	17
**p**			**0,01**	**0,01**
	**Number of patients with mucositis and neutropenia**	**Total number of CT cycles with mucositis and neutropenia**	**Average number of CT cycles with mucositis and neutropenia/patients**	**Percentage of cycles of CT with mucositis and neutropenia (%)**
**CNS Tumor**	6	9	1,5	5,5
**Sarcomas**	15	45	3	13,5
**Lymphomas**	6	16	2,7	14,5
**Other**	11	18	1,6	11,1
**p**			**0,01**	**0,01**
	**Number of patients without any episodes of mucositis**	**Total number of CT cycles without mucositis**	**Average number of CT cycles without mucositis /patients**	**Percentage of cycles of CT without mucositis (%)**
**CNS Tumor**	27	159	4,8	94
**Sarcomas**	5	278	12,1	83
**Lymphomas**	5	89	4,9	81
**Other**	5	134	7,9	83
**p**			**0,01**	**0,01**

Based on pain assessment using the WBS, we registered the following: 26 episodes, score 1 (22.6%); five episodes, score 2 (4.4%); 26 episodes, score 3 (22.6%); 25 episodes, score 4 (21.7%); and 33 episodes, score 5 (28.7%).

The episodes of mucositis were classified according to the WHO oral mucositis grading scale: 27 episodes, grade I (23.5%); 28 episodes, grade II (24.4%); 25 episodes, grade III (21.7%); and 35 episodes, grade IV (30.4%). **Table 3** summarizes the characteristics of episodes of mucositis grouped according to the score obtained using the WBS and their class in the WHO oral mucositis grading scale.

**Table 3:** Characteristics of the mucositis episodes.

WBS	Number of episodes	Average minimum number of neutrophils /episodes (cells/ml)	Percentages of episodes that needed treatment (%)	Average duration of treatment /episodes (days)
**1**	26	155	15	6
**2**	5	135	80	5,5
**3**	26	215	61,5	6
**4**	25	200	68	8
**5**	33	160	100	9
**p**		0,76	**<0,01**	0,07
**WHO oral mucositis grading scale**	**Number of episodes**	**Average number of neutrophils** **/episodes (cells/ml)**	**Percentages of episodes that needed treatment (%)**	**Average duration of treatment** **/episodes (days)**
**I**	27	110	33,3	5,5
**II**	28	220	78,6	6
**III**	25	200	100	7
**IV**	35	160	100	9
**p**		0,28	**<0,01**	**0,04**

In the first part of the table the episodes are grouped according to the score obtained in the WBS. In the second part the episodes of mucositis are grouped according to the class they belong to the WHO oral mucositis grading scale.

**Figure 2:**
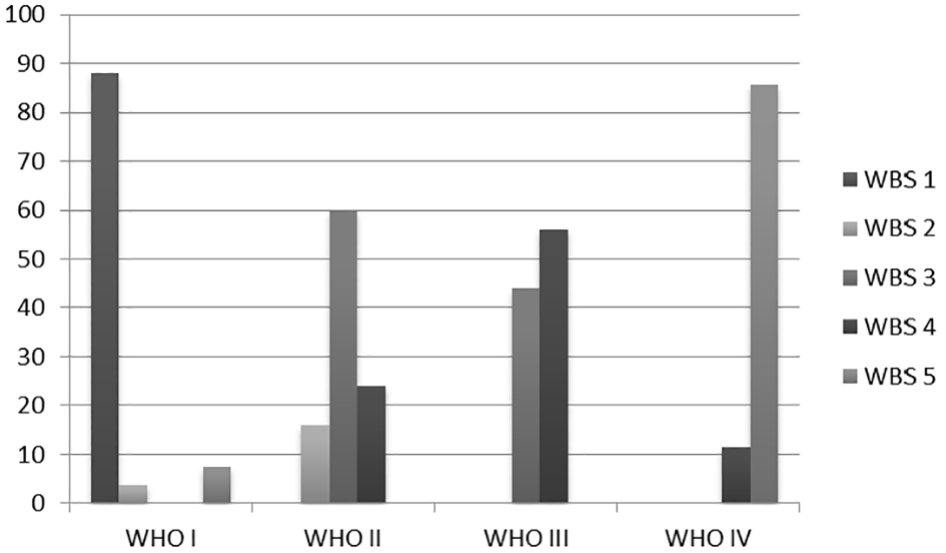
Relationship between the pain expressed by patients through the WBS and WHO oral mucositis grading scale.

Analysis of the relationship between the pain expressed by patients through the WBS and the objectivity found through the WHO oral mucositis grading scale showed the following: for WHO I, 24 episodes of 27 received a score equal to 1 of the WBS (88.89%), one episode a score of 2, and two episodes a score of 5; for WHO II, 15 of 28 episodes (53.57%) received a score of 3, six episodes a score of 4, four episodes a score of 2, and only one episode a score of 5; for WHO III, we observed a fair distribution between WBS 3 and 4, with 11 (44%) and 14 (56%) cases of 25 respectively; for WHO IV 30 of 35 cases (85.7%) had a score of 5, while the remaining five had a score of 4 (14.3%). **Figure 2** illustrates the preceding data.

**Figure 3:**
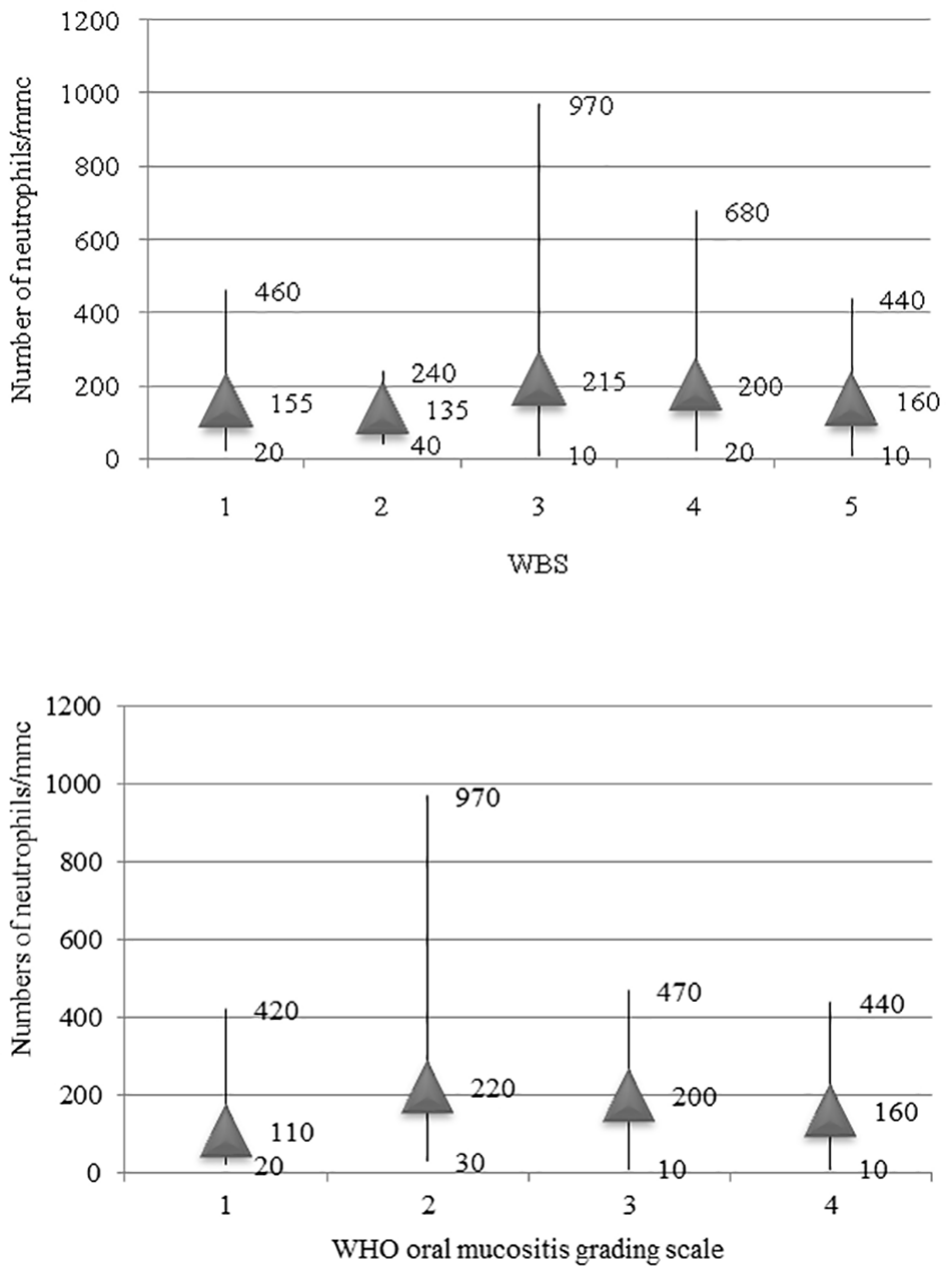
Relationship between the minimum number of neutrophils observed during the episode of mucositis and the grade of mucositis according to WBS and WHO oral mucositis scale.

Furthermore, we have evaluated the relationship between neutropenia (<500 cells/ml) and the onset of oral mucositis. Therefore, we analyzed the number of episodes of neutropenia affecting the 84 patients during the observation period (i.e., during the 775 total chemotherapy cycles administered) and the number of episodes of neutropenia associated with mucositis. The odds ratio was 20.3 (p < 0.01). A total of 78 episodes of mucositis (68%) were linked to a neutrophil count <500 cells/ml. Hence, confirmation of neutropenia is a significant risk factor for the development of this complication. There was no relationship between the neutrophil count and severity of pain assessed by the WBS or WHO oral mucositis grading scale; in fact the averages of the minimum value of neutrophils observed during the episodes of mucositis were not statistically different into the WBS class and WHO grade (**Table 2**, **Figure 3**).

In 89 episodes of mucositis (77.4%), a pain-relieving pharmacological treatment was required in addition to the mouth rinses for seven, 22, 25, and 35 cases of WHO grades I, II, III, and IV mucositis, respectively.

Pain relief was achieved in 79 episodes (68.7%) following the intravenous administration of pethidine for seven, 21, 22, and 29 cases of WHO grades I, II, III, and IV, respectively.

In 10 episodes (8.7%), fentanyl was used to support oral rinses: four and six cases of WHO grades III and IV, respectively.

Finally, 26 episodes (22.6%) did not require any additional drug supplement to the oral rinses: 20 and six cases of WHO grades I and II, respectively.

The duration of drug treatment was longer for children with WHO grades III and IV (**Table 2**). This difference was statistically significant (p = 0.04). Total parenteral nutrition was prescribed for 26 episodes of 115 (22.6%). We did not observe correlations between neutrophil count and the duration of drug treatment (r = −0.02; p = 0.87).

## Discussion

In recent years, there has been a noticeable improvement in terms of survival for children with cancer. This was achieved owing to the intensification of medical treatments which, however, led to a parallel increase in the rate of side effects (1–3, 19).

Mucositis represents one of the most frequent side effects in children receiving chemotherapy with an incidence ranging 52–100% of the cases described in the literature (17). This finding was confirmed in our population, in which we diagnosed mucositis in 50% of the patients.

Recent studies have reported a difference in the prevalence of mucositis in relation to sex. However, these data are not homogeneous as some investigators reported a female predominance (20), whereas others noted a male predominance (21). In our population, there was no difference in relation to sex observed; hence, it appears that sex is not a factor influencing the occurrence of mucositis.

Although a correlation between age and the number of episodes of mucositis was not detected in our patients (21), the incidence of mucositis was significantly higher in children aged >10 years. We hypothesized that this result is linked to the lower ability for oral mucosa renewal in older children (22) and the less attention paid by adolescents to oral hygiene.

On average, mucositis episodes occurred 10.4 days after the initiation of chemotherapy and lasted 7.3 days, as recently demonstrated also by Hurrell et al. (23).

According to the diagnosis, patients were stratified into four groups: patients with central nervous system tumors; patients with sarcomas; patients with lymphomas; and patients with the remaining solid tumors, which mainly included neuroblastomas, nephroblastomas, and retinoblastomas. We observed a greater number of episodes of mucositis in patients with sarcomas or lymphomas. This relationship may be linked to the correlation between the number of cycles of chemotherapy performed and episodes of mucositis observed (r = 0.52; p < 0.01), as well as the different antineoplastic agents used in the respective treatment protocols. The use of polychemotherapy did not allow us to identify the drugs most frequently implicated in the onset of mucositis. However, methotrexate and anthracyclines are frequently utilized for the treatment of lymphomas and sarcomas. These two chemotherapeutic agents are among the drugs with the greatest risk of causing mucositis. This was confirmed by the higher incidence of mucositis in patients receiving chemotherapy for leukemia, for whom treatment with anthracyclines and methotrexate is essential, as observed in previous studies (19–24).

Furthermore, lymphomas and sarcomas are the two types of cancer in which we observed the highest prevalence of neutropenia-associated mucositis. Neutropenia is an established risk factor for the development of mucositis (25, 26). This has also been confirmed by our investigation, which showed an odds ratio equal to 20.3 (p < 0.01) for neutropenia. The presence of neutropenia, linked to more intensive therapeutic treatments, is certainly an important factor influencing the higher prevalence of mucositis recorded in the group of patients with sarcomas and lymphomas. However, in our study we included only patients undergone to standard chemotherapy and not to high-dose chemotherapy. For this reason we cannot be sure that the degree of neutropenia will not affect the severity and duration of mucositis in the case of high-dose chemotherapy with more intense neutropenia.

By adopting the WHO oral mucositis grading scale, we observed that all episodes of mucositis classified as more serious (classes III and IV) required pain relief treatment through the intravenous route. Furthermore, the duration of treatment for these episodes was significantly longer than that for less serious episodes (classes I and II) (p = 0.04). Therefore, use of the WHO oral mucositis grading scale is effective in adequately assessing the severity of episodes of mucositis in children (27).

The WHO oral mucositis grading scale provides an in-depth assessment of the oral cavity. However, in pediatric patients, this is not always possible due to poor compliance. Therefore, we attempted to analyze the possible correspondence between the WHO oral mucositis grading scale, an objective and integrative assessment scale of signs and symptoms, with a pain assessment scale widely used and validated in children, such as the WBS (19). The WBS is easy to apply in pediatric patients, as it allows the collection of data relating to the discomfort experienced by the patient without the need to resort to an objective evaluation, which is not always obtainable in this group. Moreover, it can be easily repeated several times during the same day to adequately modulate therapy. Similar to the WHO oral mucositis grading scale, the episodes described as more serious directly by the patient were those that required intravenous drug treatment. Furthermore, we observed an almost total overlap between the degree of pain described by the patients and the objectivity found during the evaluations performed using the WHO oral mucositis grading scale. This allows us to conclude that, in patients in whom an objective assessment of the degree of mucositis is not possible, application of the WBS may be sufficient in guiding the choice of intravenous treatment.

As previously mentioned, the presence of neutropenia represents a risk factor for the development of mucositis. However, we observed that the degree of neutropenia does not affect the severity of the mucositis episode and does not influence the duration of treatment (r = −0.02; p = 0.87).

Although the data in the literature are often discordant and not unequivocal in interpretation, the importance of adequate oral hygiene as a protective factor for the development of mucositis appears to be definitive. The use of specific oral hygiene protocols for prophylactic purposes associated with a continuous education intervention performed by health personnel, though not preventing the onset of mucositis, has proved to be fundamental in reducing its duration and severity (28).

In the study population, the oral hygiene protocol was respected in almost 100% of cases, which allowed for a prevalence of mucositis equal to 50%, slightly lower than the rates previously described in the literature (12).

In 89 episodes of mucositis (77.4%), a pain-relieving pharmacological treatment was required in addition to the mouth rinses; in 79 episodes (68.7%), intravenous administration of pethidine was sufficient, while in 10 episodes (8.7%), patients needed more intensive drug treatment with intravenous fentanyl through continuous infusion. These 10 episodes belonged to classes III and IV of the WHO oral mucositis grading scale, confirming the effectiveness of this evaluation system in predicting the intensity of the treatment to be adopted.

Therefore, therapy with intravenous pethidine was effective in 68.7% of cases, and only 8.7% necessitated more intense drug treatment, such as intravenous fentanyl. During patient observation, therapy with pethidine was absolutely safe and did not lead to any side effects. Thus, patients perfectly tolerated and responded promptly to the treatment. This confirms the findings of a previous study conducted by Oudot et al. (29), which revealed that the use of pethidine demonstrated the same efficacy as that of major opioid drugs, but with fewer side effects.

Only 26 cases of the 115 analyzed (22.6%) required parenteral feeding. In fact, the severity and duration of mucositis were such that the use of pethidine, fentanyl, and oral rinses was insufficient to improve the functionality of the oral cavity and allow the patient to quickly recover the ability to independently drink and eat.

## Conclusion

Oral mucositis is a common complication of chemotherapy for childhood malignancies, which has an important influence on patient quality of life and compliance to treatment. In our study, 50% of the patients experienced at least one episode of mucositis during the treatment period. Moreover, the factors that affected its occurrence are the presence of neutropenia, the number of cycles of chemotherapy performed, and the type of tumor.

The WHO oral mucositis scale is a valuable tool for assessing the severity of mucositis in pediatric patients and the WBS can be used as an assessment tool for establishing the therapy to be adopted in patients in whom direct evaluation of the severity of mucositis is not possible.

The treatment currently used in our clinical practice is effective and safe, without significant adverse events recorded.

## Data Availability Statement

The raw data supporting the conclusions of this article will be made available by the authors, without undue reservation.

## Ethics Statement

Prior to their participation, the caregivers provided signed consent forms after being informed about the aim of the project as foreseen by the Italian Law on Privacy and the Safeguarding of Sensitive Data (D.Lgs n196, 2003). The project was performed in accordance with the principles of the Declaration of Helsinki. Approval by the ethics committee was not necessary for a retrospective observational study on clinical practice data.

## Author Contributions

Conceptualization, ARu, ARo, and GA. Methodology, ARu and PM. Writing—original draft preparation, ARo, MC, and ST. Writing—review and editing, SM and AD. Supervision, ARu. All authors contributed to the article and approved the submitted version.

## Conflict of Interest

The authors declare that the research was conducted in the absence of any commercial or financial relationships that could be construed as a potential conflict of interest.
